# Improved Auditory Nerve Survival with Nanoengineered Supraparticles for Neurotrophin Delivery into the Deafened Cochlea

**DOI:** 10.1371/journal.pone.0164867

**Published:** 2016-10-27

**Authors:** Andrew K. Wise, Justin Tan, Yajun Wang, Frank Caruso, Robert K. Shepherd

**Affiliations:** 1 The Bionics Institute, 384–388 Albert Street, East Melbourne, Melbourne, Australia; 2 The Department of Medical Bionics, University of Melbourne, Melbourne, Australia; 3 Department of Otolaryngology, University of Melbourne, Melbourne, Australia; 4 ARC Centre of Excellence in Convergent Bio-Nano Science and Technology, and the Department of Chemical and Biomolecular Engineering, the University of Melbourne, Melbourne, Australia; University of Washington, UNITED STATES

## Abstract

Cochlear implants electrically stimulate spiral ganglion neurons (SGNs) in order to provide speech cues to severe-profoundly deaf patients. In normal hearing cochleae the SGNs depend on endogenous neurotrophins secreted by sensory cells in the organ of Corti for survival. SGNs gradually degenerate following deafness and consequently there is considerable interest in developing clinically relevant strategies to provide exogenous neurotrophins to preserve SGN survival. The present study investigated the safety and efficacy of a drug delivery system for the cochlea using nanoengineered silica supraparticles. In the present study we delivered Brain-derived neurotrophic factor (BDNF) over a period of four weeks and evaluated SGN survival as a measure of efficacy. Supraparticles were bilaterally implanted into the basal turn of cochleae in profoundly deafened guinea pigs. One ear received BDNF-loaded supraparticles and the other ear control (unloaded) supraparticles. After one month of treatment the cochleae were examined histologically. There was significantly greater survival of SGNs in cochleae that received BDNF supraparticles compared to the contralateral control cochleae (repeated measures ANOVA, p = 0.009). SGN survival was observed over a wide extent of the cochlea. The supraparticles were well tolerated within the cochlea with a tissue response that was localised to the site of implantation in the cochlear base. Although mild, the tissue response was significantly greater in cochleae treated with BDNF supraparticles compared to the controls (repeated measures ANOVA, p = 0.003). These data support the clinical potential of this technology particularly as the supraparticles can be loaded with a variety of therapeutic drugs.

## Introduction

Hearing loss is one of the most common sensory deficits affecting over 5.3% of people worldwide (approximately 360 million people: World Health Organization, 2012). Deafness can have a significant impact on communication in a hearing world and affect the development of language in children [1], with social, vocational and mental health implications throughout life [2]. The number of people impacted by deafness is expected to rise as the population ages.

Sensorineural hearing loss (SNHL) is the most common form of deafness and typically results from damage to the delicate sensory hair cells within the cochlea, or loss of their synaptic connections with the spiral ganglion neurons (SGNs). The only therapeutic option for people with profound to severe SNHL is a cochlear implant; a bionic device that restores hearing function via electrical stimulation of the remaining SGNs to effectively bypass the lost sensory modality.

However, one major consequence of SNHL is the progressive degeneration of the SGNs that occurs in both humans [3, 4] and animal deafness models [5–7]. A primary cause of SGN degeneration is the loss of the endogenous supply of neurotrophins, in particular Brain-Derived Neurotrophic Factor (BDNF) and Neurotrophin 3 (NT-3), which are normally produced by hair cells and supporting cells in the organ of Corti [8–13]. Both BDNF and NT-3 are required for normal neural development and innervation of cochlear hair cells with NT-3 particularly important for synaptogenesis [14, 15]. As the SGNs are the target neurons for the cochlear implant a functional population of SGNs is essential for the implant to perform effectively. For example, a recent study has shown a strong correlation between word recognition scores and SGN counts indicating that a greater population of SGNs results in better cochlear implant performance [16]. As such, clinically relevant strategies that can prevent the progressive degeneration of SGN with cochlear implantation have received substantial interest.

Exogenous administration of neurotrophins using implantable mini-pumps has been shown to improve the survival of SGNs in animal deafness models [17–24] with the SGN peripheral fibres becoming larger and more numerous compared to untreated cochleae [20, 25]. However, the supply of neurotrophins from pump-based devices is finite, necessitating the need for pumps to be refilled or replaced and thus leading to concerns over the long-term safety of these devices as a result of infection [26]. Although the long-term survival-promoting effects following the cessation of exogenous neurotrophin delivery are yet to be established there is some evidence that improved SGN survival may persist for at least a short period of time (weeks) following the removal of the exogenous neurotrophin supply [27, 28]. However, this is not a universal finding with one previous study showed no sustained survival effect following cessation of neurotrophin delivery [18]. It is therefore likely that long-term neurotrophin delivery strategies will be required for the survival effects to be maintained over time. Encouragingly, SGN survival can be enhanced when exogenous neurotrophins are applied in concert with electrical stimulation from a cochlear implant (with SGNs also exhibiting lower electrical thresholds) [29–32] meaning that even at low levels, neurotrophin delivery might remain therapeutically beneficial when combined with cochlear implant use.

Although there are potential benefits of neurotrophin therapy for clinical application a significant factor limiting translation is the need for a drug delivery mechanism that provides a long-term supply of the neurotrophins in a safe and effective manner. Given the potential infection risks of implantable pump devices [26] and the limitations of systemic delivery in crossing the blood-labyrinth barrier, research has focused on localised neurotrophins delivery strategies that are clinically translatable and that can be combined with a cochlear implant. These include cell-based therapies [32–34], gene therapies [35–41], electrode coating materials [42–45] and biocompatible carrier systems including nanoparticles [46–49]. Although each approach has advantages and limitations, the use of nanoengineered carriers offers promise of delivering therapeutic levels of neurotrophins in a safe manner that is effective over the long term.

Recently we have developed a new drug carrier system that uses mesoporous silica nanoparticles as building blocks to form larger (~ 500 μm) supraparticles (SPs) [50]. *In vitro* experiments showed that the SPs could be loaded with high payloads of BDNF and a pilot *in vivo* experiment showed that BDNF loaded SPs could be surgically implanted into the cochlea of the guinea pig.

In the present study we have evaluated the efficacy of BDNF-loaded SPs to protect SGNs from deafness-induced degeneration and assessed the biocompatibility of the SP carrier system by quantifying the tissue response following chronic implantation. The results showed that SPs were biocompatible and able to deliver a therapeutic level of BDNF that resulted in significant SGN survival that was observed throughout a wide extent of the cochlea compared to deafened control cochleae. This finding highlights the clinical suitability of the supraparticle system that can overcome some of the significant safety concerns of pump-based drug delivery devices yet yield similar therapeutic effects in terms of the level and extent of SGN survival throughout the cochlea, an outcome that has proven difficult to achieve in other drug delivery strategies.

## Materials and Methods

### Experimental Animals

Six young adult pigmented guinea pigs of either sex (mean 550 g) were used in this study. Both ears were used resulting in a total of 12 cochleae. All procedures were approved by the St. Vincent’s Hospital Animal Research & Ethics Committee (ref# 014/12-r1) in accordance with the National Institutes of Health (NIH) Guidelines for the Care and Use of Laboratory Animals, and conformed to the Code of Practice of the National Health and Medical Research Council of Australia.

### Auditory brainstem responses

Only animals with otoscopically normal external ears were used in this study. Prior to any surgical procedure the hearing status was assessed under anesthesia (ketamine, 60mg/kg (Parnell Australia) and xylazine, 4mg/kg (Ilium, Australia); intramuscular injection). Click-evoked auditory brainstem responses (ABRs) were measured to assess the hearing status of the guinea pigs before and after deafening using procedures described previously [20, 35]. The anaesthetized guinea pigs were placed on a heat pad with the temperature maintained at 37°C in a sound attenuated room and needle electrodes were placed at the skull vertex, at the nape of the neck and on the abdomen. ABRs were measured to acoustic clicks delivered by a calibrated speaker at intensities up to 100 dB peak equivalent (p.e.) sound pressure level (SPL). The ABR was amplified, recorded to computer and averaged over 200 trials that were presented at stimulus intensities ranging from 0 dB to 100 dB p.e. SPL. Hearing threshold was determined and only guinea pigs with normal hearing thresholds in both ears (ABR threshold <50 dB p.e. SPL) were used in the study.

### Deafening Procedure

Animals were deafened ototoxically using a procedure which has been previously shown to produce a severe bilateral SNHL [30, 35, 51]. Under gaseous anesthesia (1–2% isoflurane in O_2_, 1L/min) the right jugular vein was exposed and cannulated. Frusemide (130mg/kg; Ilium, Australia) diluted in warm Hartmann’s solution was slowly injected. The vein was tied off and the incision sealed with cyanoacrylate. The ototoxic aminoglycoside kanamycin sulfate (420mg/kg; Sigma-Aldrich, USA) dissolved in 3ml Hartmann’s solution was then injected subcutaneously (s.c.). Approximately four days after the deafening procedure ABRs were re-measured as described above, to confirm deafness. An increase in hearing thresholds of >50dB indicated severe to profound SNHL. All thresholds recorded were >100dB p.e. SPL, which was the maximum intensity presented.

### Supraparticle Preparation

The SPs were synthesized as described previously [50]. Briefly, mesoporous silica nanoparticles (diameter, ca. 400 nm) [52] were dispersed in water at a concentration of 5 wt% and formed a stable colloidal suspension. Droplets (0.5 μl) of the suspension solution were applied to a hydrophilic surface (paraffin film) before being dried with air flow. Under capillary force, the mesoporous silica colloids self-assemble into a compact structure to form SPs. The SPs were then annealed at 923°K to enhance mechanical stability of the SPs. The SPs had a bimodal pore structure (~2–3 nm and 15–30 nm) and the space within the SP, between the densely packed nanoparticles, was ~100–200 nm, resulting in a porous structure with a high surface area. The SPs used in this study had a diameter of ca. 500 μm.

### Supraparticle sterilization, NT loading and NT release properties

Supraparticles were sterilized by soaking them in 100 μL of ethanol (80 vol/vol%) for 4 hours prior to rinsing with 100 μl of Milli-Q water six times. SPs were then washed once in 0.1 M phosphate buffered saline (PBS). Eight SPs were loaded for each experimental animal by placing them in an Eppendorf tube containing 15 μl of BDNF (Geneway, BDNF Human Protein, Cat. # 10-663-45078) solution (1 mg/ml of BDNF) and incubating them at ambient room temperature for three days with occasional shaking. Immediately prior to implantation the BDNF-loaded SPs (BDNF-SPs) were rinsed once in 20 μl of Milli-Q water and placed in sterile saline prior to implantation. The SPs possess large pore volumes and high surface areas, and therefore allow large amounts of BDNF to be loaded. In previous *in vitro* elution studies we have reported that each SP could absorb approximately 1.3 μg of BDNF during the loading procedure and approximately 400 ng of BDNF could be released via a linear release profile over the first month [50]. The porous characteristics of the supraparticles are thought to improve the bioeffectiveness of the BDNF by immobilizing the protein and limiting its breakdown in the *in vivo* environment [53, 54].

### Bilateral implant surgery

**Fig 1:**
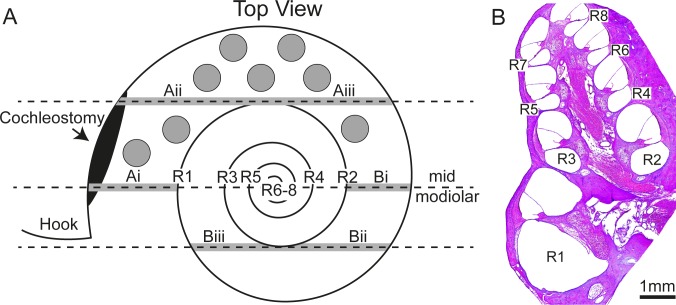
Schematic illustration of the top view looking down on the cochlea depicting the approximate location where the SPs were implanted (grey circles). The cochlear regions that were analysed for SGN density (region 1 to region 8; R1-R8) and tissue response to implantation (grey region on dotted lines–Ai-iii and Bi-iii) are shown. Tissue response data was averaged across region A and B. (B) A mid modiolar micrograph of an implanted cochlea showing the cochlear regions in which SGN density within Rosenthal’s canal was analysed (R1-R8).

One week following deafening the animal underwent bilateral cochlear implant surgery using aseptic surgical techniques. Guinea pigs were anaesthetized with an intramuscular injection of ketamine (60 mg per kg body weight) and xylazine (4 mg per kg body weight). Using a post-auricular approach, the bulla was exposed and a small hole was drilled to expose the basal turn of the cochlea. A cochleostomy (approximately 800 μm) was made using a 0.8 mm diamond drill and gentle suction was applied to remove bone debris from the cochlea (Fig 1). A total of 8 SPs were placed into the basal turn of the cochlea using a 21 gauge polyurethane catheter (Optiva, Medex Medical, UK). One cochlea was implanted with BDNF-SPs and the other cochlea was implanted with unloaded SPs (control-SPs) using identical surgical techniques. The side implanted with the BDNF-SPs was randomized. Following implantation, a small piece of muscle was placed over the cochleostomy in order to seal the cochlea. The wound was closed by suturing surrounding muscles in two layers and closing the skin incision with staples. Hartmann’s solution (10ml/kg; s.c.), the antibiotic Baytril (Bayer, Germany) (0.10mg/kg; s.c.), and the analgesic Temgesic (Reckitt-Benckiser, UK) (50 mg/kg; s.c.) were given after surgery and on the next day to aid recovery.

### Tissue collection and preparation

Following the 28 day treatment period, the animals were given an overdose of pentobarbital (150mg/kg; intraperitoneal) and transcardially perfused with 0.9% NaCl (37°C) followed by 10% Neutral Buffered Formalin (10% NBF; 4°C). The cochleae were dissected and a small hole was made in the apex. The round and oval windows were incised with a 25 gauge needle and the cochleae post-fixed in 10% NBF for one hour on a shaker at room temperature. The cochleae were then placed in 10% ethylenediamine tetraacetic acid (EDTA) in PBS at room temperature for decalcification. After decalcification, cochleae were cryo-protected in 15% and then 30% sucrose solution before they were embedded in Tissue-Tek O.C.T. cryosectioning compound (Sakura, Japan) as described previously [51] and stored at -80°C. Cochleae were sectioned at 12μm using a CM 1900 UV cryostat (Leica, Germany) at -22°C in the modiolar plane and mounted onto Superfrost-Plus slides (Menzel-Gläser, Braunschweig, Germany). A representative series of cochlear sections were stained with Mayer’s haemotoxylin and Putt’s eosin (H&E) for general qualitative examination, SGN density measurements within Rosenthal’s canal and quantification of the tissue response.

A representative series of mid modiolar sections were also processed for immunohistochemistry. Standard immunofluorescent protocols were followed using antibodies against heavy chain neurofilament (NF-H; Merck Millipore, Australia) to stain the SGNs and peripheral fibres using AlexaFluor secondary antibodies for visualisation (Molecular Probes, USA). Slides were mounted in media containing DAPI and imaged on a Zeiss Axioplan fluorescence microscope (Carl Zeiss, Germany). Peripheral nerve fibres located in the osseous spiral lamina in the upper basal turn (R2) were imaged from four non-consecutive mid modiolar sections (72 μm separation). Images were analysed in ImageJ V1.46 (NIH, USA, http://rsb.info.nih.gov/ij/index.html) by thresholding neurofilament labelled pixels located using the inbuilt “moment” threshold filter [32]. Nerve fibres were quantified by determining the area of thresholded pixels located within the osseous spiral lamina to obtain a percentage value. Data was averaged across the four sections.

### Spiral ganglion neuron density measurement

All quantification was carried out by a single observer blinded to the experimental groups. Sections were viewed and imaged using an Axio Lab microscope and software (Zeiss, Germany). In the hematoxylin and eosin-stained sections the area of Rosenthal’s canal was measured for each cochlear region (Region 1 to 8; see Fig 1) using ImageJ V1.46. SGNs were identified within Rosenthal’s canal and counted. Only SGNs exhibiting a clear nucleus were counted using previously published techniques [32, 33, 51, 55, 56]. Data was collected from five non-continuous sections with a separation of 72 μm ensuring that no cell was counted more than once. The density of the SGNs was determined for each cochlear region as an averaged from five sections. In the apical cochlear regions (regions 6–8) Rosenthal’s canal typically narrows and therefore SGN density data for regions 6–8 were combined.

### Tissue response measurements

In order to quantify the immune response to the implanted SPs, the extent of fibrosis and new bone growth in the cochlea was measured in hematoxylin and eosin-stained sections at three different locations separated by a distance of at least 400 μm. The tissue response was quantified as the percentage of the area of the scala tympani occupied by any inflammatory cells or fibrous tissue and new bone using techniques previously described by [32] and similar to those implemented by [57, 58]. Briefly, an image of the scala tympani was captured and the area measured. The ‘Moments’ algorithm in Image J was used to threshold the image to identify the tissue response. The area of scala tympani was measured and the proportion of the scala tympani occupied by the tissue response was calculated. Data was measured from a total of six positions within the cochlea and with three data points per cochleae averaged for the lower basal region (Ai-Aiii) and three data points averaged for the upper basal region (Bi-Biii) (see Fig 1A).

### Statistics

Statistical comparisons of SGN density and tissue response were made by comparing data from the cochleae implanted with BDNF-SPs to the data in the contralateral control cochlea that were implanted with control-SPs. A repeated measures (RM) analysis of variance (ANOVA) using p<0.05 level of significance was used to determine statistical significance and post hoc comparisons were made using the Holm Sidak method.

## Results

### Spiral ganglion neuron survival

**Fig 2:**
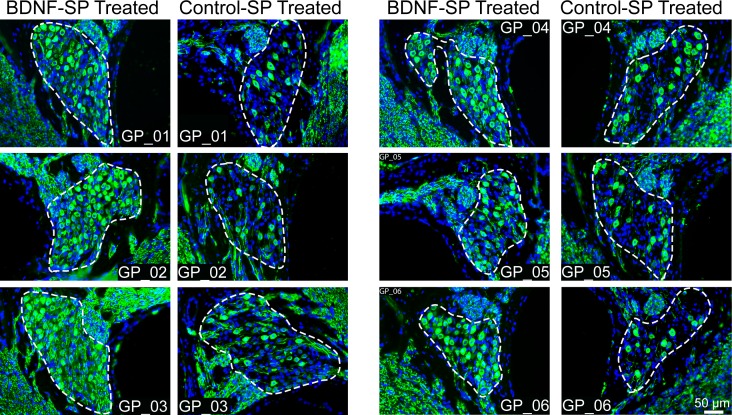
Example cochlear sections of the BDNF-SP and Control-SP treated cochleae for all six animals used in this study (GP_01 to 06). Images show the upper basal turn (region 2) and sections are stained with a neuronal marker (neurofilament: green) and a nucleus marker (DAPI: blue). Rosenthal’s canal containing the SGN is depicted by the white dotted line in each image. It is clearly evident that there is greater SGN survival in the BDNF-SP treated cochleae compared to the contralateral cochleae that received Control-SP treatment. This is consistent across the entire cohort of animals used in the study.

Mid modiolar cochlear sections were stained with a neuronal antibody (neurofilament) that specifically labels SGNs and their nerve fibres. Example data from all of the cochleae used in this study is shown in Fig 2. Images show the SGNs within Rosenthal’s canal (dotted lines) in the upper basal turn (region 2) for each guinea pig (GP_01 to 06 labeled within each image). There is a clear difference in SGN survival with the cochleae that received BDNF-SP exhibiting more SGNs compared to the contralateral cochleae that received Control-SP. To ascertain the effects of BDNF-SP treatment on peripheral fibre survival analysis of the peripheral nerve fibres in the upper basal region (region 2) was carried out by determining the proportion of the osseous spiral lamina occupied by the nerve fibres. The nerve fibres occupied a greater proportion of the osseous spiral lamina in cochleae implanted with BDNF-SPs (19.1% ± 1.3 SEM) compared to control-SPs (13.6% ± 1.3 SEM) (two tailed paired t-test, p = 0.002).

**Fig 3:**
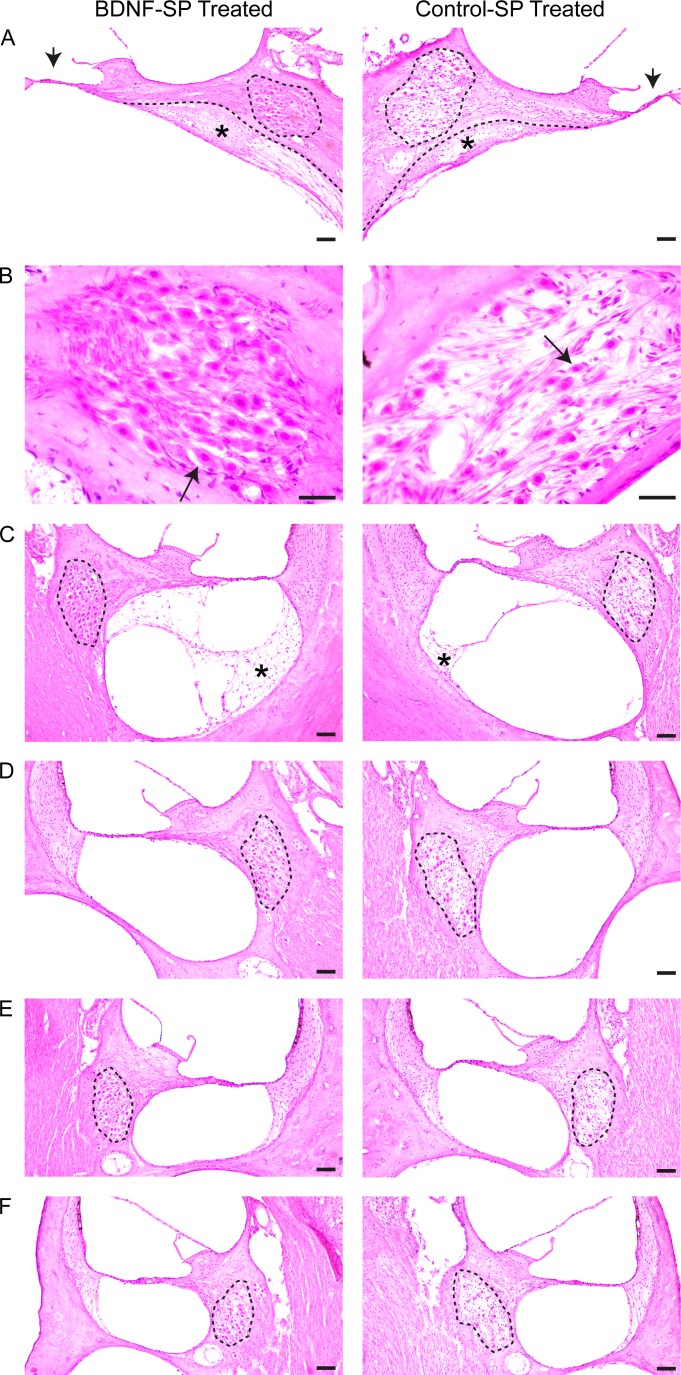
Representative examples of cochlear sections obtained from a cochlea treated with BDNF-SPs (left) and Control-SPs (right) for cochlear Regions 1–5 (A-F respectively). (A) shows the lower basal region (Region 1) with Rosenthal’s canal outlined (dotted outline). There was a flattening of the sensory epithelium and complete loss of the organ of Corti (arrow) that was symmetrical between ears. There was a tissue response (*; Region Ai) to the presence of the SPs that was observed in the scala tympani with the osseous spiral lamina depicted with a dotted line. (B) Higher magnification image of SGNs (arrows) in Rosenthal’s canal in Region 1 from BDNF-SP treated cochlea (left) and from a Control-SP treated cochlea (right). There was a greater density of SGNs in the BDNF-SP treated cochlea compared to the control cochlea. (C) Cochlear section taken at Region 2 shows the Rosenthal’s canal (dotted line) and the fibrotic tissue response (*; Region Bi) for the BDNF-SP (left) and Control-SP (right) cochleae. (D–F) Cochlear section taken at Region 3–5 shows the Rosenthal’s canal (dotted line) for the BDNF-SP (left) and Control-SP (right) cochleae. There was no tissue response in these cochlear regions. Scale bar (A and C-F = 100 μm) and (B = 50 μm).

A representative series of cochlear sections were stained with hematoxylin and eosin to quantify the effect of BDNF-SP treatment on SGN survival. Fig 3 shows representative examples of cochlear regions 1–5 (R1-5) taken from a cochlea implanted with BDNF-SPs (left panel) and from the contralateral control cochlea implanted with Control-SPs (right panel). Aminoglycoside deafening resulted in bilateral threshold shift of >50dB to acoustic clicks (data not shown) and complete loss of the organ of Corti throughout the cochlea (Fig 3).

In order to quantify SGN survival the density of SGNs was calculated by measuring the area of Rosenthal’s canal (dotted lines in Fig 3) and counting the number of SGNs. Fig 3A and 3B shows a section taken from the lower basal turn (region 1) for a BDNF-SP treated (left) and Control-SP treated (right) cochlea at low (A) and higher magnification (B) of the SGNs. There was a significantly greater density of SGNs in cochleae that were treated with BDNF-SPs compared to the Control-SP cochleae. Importantly, the greater SGN survival was evident throughout the cochlea as can be seen in Fig 3C to 3F showing representative data from cochlear regions 2–5.

**Fig 4:**
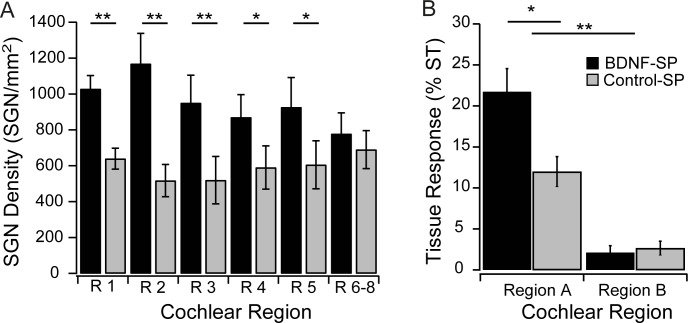
(A) Average of the SGN density measured in each cochlear region after one month of treatment with BDNF-SP (black) and the Control-SPs (Grey). There was a significantly greater density of SGNs in cochleae treated with BDNF-SPs compared to the Control-SPs (two way ANOVA, p = 0.009) with post hoc analysis indicated (Holm-Sidak, **p<0.005, *p<0.05). Error bars ± 1 SEM. (B) Analysis of the cochlear tissue response measured in the scala tympani (ST) in cochlear Regions A and B showed a tissue response for Region A (near the site of the cochleostomy) that was significantly larger than the tissue response in Region B (Post Hoc Holm-Sidak; **p<0.001). The tissue response measured in Region A in the BDNF-SP treated cochleae was greater than that in the Control-SP treated cochlea (Post Hoc Holm-Sidak; *p = 0.003).

Analysis of the SGN density data showed a statistically greater density of SGNs in the cochleae treated with BDNF- SPs (two way repeated measures ANOVA, p = 0.009) using ‘cochlear region’ and ‘neurotrophin treatment’ as factors. Post hoc analysis indicated that a significant difference in SGN density was observed in all cochlear regions except the most apical regions (region 6–8) (Holm-Sidak, **p<0.005, *p<0.05; Fig 4).

### Tissue Response

A prerequisite for the clinical viability of the SP drug delivery system is that it is safe for implantation in humans. In order to determine SP safety the foreign body tissue response was measured within the cochlea (see Fig 1). Fig 3 shows examples of the tissue response throughout the cochlea to the implantation of the SPs. The tissue response was restricted to the scala tympani near the site of implantation in the basal cochlear region (region A and B; see Fig 1). The tissue response was made up of loose fibrotic tissue, small areas of osteoid or bone, with the occasional presence of active inflammatory reaction as indicated by the presence of lymphocytes neutrophils and multinucleated giant cells.

The tissue response was quantified by measuring the proportion of the scala tympani occupied by the new tissue and the data is presented in Fig 4B. The tissue response was most prominent in the scala tympani Region A, in close proximity to the cochleostomy and the location of the SPs within the cochlea. There was a significant interaction between cochlear Region and SP Treatment (two way repeated measures ANOVA, p = 0.029). Post hoc analysis indicated that the tissue response was significantly greater in Region A compared to Region B (Holm-Sidak, p<0.001) and was significantly greater in the cochleae implanted with the BDNF-SPs compared to the cochleae implanted with the Control-SPs (Holm-Sidak, p = 0.003).

## Discussion

This study examined the safety and efficacy of intracochlear implantation of mesoporous silica SPs that were designed as a carrier for BDNF delivery in the inner ear. We implanted BDNF-SP or Control-SPs into the basal turn of deafened guinea pigs and, following a four week treatment period, examined the cochleae histologically to measure SGNs survival and SP biocompatibility. The results showed significant SGN survival along a wide extent of the cochleae that were treated with the BDNF-SPs compared to the contralateral cochleae treated with Control-SPs. There was a significantly greater density of peripheral fibres as indicated by a greater proportion of nerve fibres within the osseous spiral lamina in the BDNF-SP treated cochleae. The SPs were implanted via a cochleostomy and this procedure resulted in a tissue response that was largely localised to the basal turn, close to the site of the cochleostomy. However, there was a small but significant increase in the tissue response in cochleae that received BDNF-SP compared to Control-SPs.

### Effects of Deafness

Animals were deafened with the aminoglycoside kanamycin and the loop diuretic frusemide. All animals were profoundly deaf and exhibited ABR thresholds over 100 dB SPL p.e. (data not shown). Histological analysis confirmed that the organ of Corti had completely collapsed, indicating that, similar to previous studies, the deafening procedure resulted in complete loss of hair cells and degeneration of the supporting cells, such that there was typically a completely flattened epithelium (Fig 3) [18, 20, 30, 35, 37, 59]. The effects of this deafening technique are symmetrical with SGN loss similar between ears [60] indicating that the contralateral cochlea serves as a reliable control for experimental techniques designed to improve SGN survival.

### Effects of BDNF-SPs

SGN survival was observed throughout a wide spatial extent of the cochlea in all but the most apical cochlear regions with SGN densities that were consistent with neural densities in normal hearing guinea pigs from previous studies [20, 30]. Many of the other clinically translatable approaches to deliver neurotrophins to the cochlea described above (e.g. gene therapy, cell-based therapy, electrode coatings and hydrogels placed on the round window) have observed SGN survival effects typically restricted to the basal cochlear regions, in close proximity to the drug delivery device. The results of the current study showed that there was no significant difference in SGN survival between BDNF-SPs and Control-SPs in the apical cochlear region (region 6–8). It is known that SGNs in the apical cochlear regions are less susceptible to degeneration following aminoglycoside exposure, even when there is complete destruction of the organ of Corti, indicating that apical SGNs are less susceptible to deafness-induced degeneration [61]. Therefore, a lack of a statistical difference in SGN survival in apical regions does not necessarily indicate that a therapeutic level of BDNF was not present. Nevertheless, the results from this study provide direct evidence that a therapeutic level of BDNF was present in the basal to upper middle cochlear regions as demonstrated here leading to an improved SGN survival in these regions.

### Biocompatibility of Supraparticles

In order to determine the safety of the SP for drug delivery the tissue response to implantation of BDNF-SPs and Control-SPs was quantified. The tissue response was primarily located in the lower basal turn (Region A), principally around the site of the cochleostomy and was restricted to a small proportion of the scala tympani. Further along the cochlea, in the middle turns (Region B) the tissue response, if present, occupied an even smaller proportion of the scala tympani. The tissue response was only observed in the scala tympani, with the other cochlear compartments (the scala vestibuli and scala media) devoid of any tissue response. These finding provide evidence that the SPs were well tolerated *in vivo*. There was a significantly greater tissue response in Region A of the BDNF-SP treated cochleae compared to the Control-SP treated cochleae. An enhanced tissue response following intracochlear BDNF delivery has been reported before [30, 62] and may reflect the use of human BDNF protein delivered to the guinea pig cochlea in this and previous studies. The extent of tissue response measured in the Control-SP cohort was consistent with that observed in previous studies that have performed a cochleostomy in the basal turn [38, 57] where the surgical exposure and bone dust generated created a local tissue response consisting of new bone growth and loose fibrotic tissue.

We were unable to obtain histological evidence of the presence of the mesoporous silica SPs within the cochlea. It is likely that the SPs remained present at the time of tissue harvesting but could not be detected in our sections due to the histological processing of the tissue (decalcification, cochlear infiltration, freezing, sectioning and washing). Our *in vitro* studies confirm that the SPs remain viable for over 70 days in saline [50].

The longer-term fate of the SPs *in vivo*, when implanted into the cochlea, is still to be determined. The SPs will eventually breakdown and be cleared by the body. The SPs are comprised of colloidal silica, which is water soluble, easily absorbed and rapidly excreted. Colloidal silica is used clinically as a dietary supplement. *In vitro* studies have shown that silica particles have no or very low cytotoxic effects in various cell lines [63]. *In vivo* experiments have shown that silica nanoparticles are biocompatible, biodegradable, and bioexcretable [64, 65] and do not affect cochlear function even when used as an intracellular carrier for hair cells and SGNs [66]. It is likely that the dissolved silica will be cleared via the cochlear vasculature. The FDA has recently approved the human trial of silica nanoparticles that are used for imaging lymph nodes in cancer patients (NCT02106598; ClinicalTrials.gov).

### Advantages of Supraparticle Carriers

Previous research has shown that nanoengineered particles (~2 μm in size) could load and deliver BDNF over a sustained duration [46]. The current study has used a SP carrier system with characteristics that make it uniquely suited for drug delivery to the cochlea. Firstly, the relatively large size of the SPs (~500 μm) meant that they were easily handled during surgical implantation into the cochlea and would ensure that they could not disperse far from the implantation site. Dispersal of smaller particles or cells from the cochlea to the central nervous system and/or the vestibular end organs is problematic because it reduces the therapeutic load to the target SGNs within the cochlea. A number of researchers have used hydrogels or foams to minimise the dispersal of particles or cells from the cochlea [46, 67]. Secondly, the SPs provide sustained released of BDNF [50] meaning that longer durations of drug delivery are possible. Thirdly, the SPs can be loaded with high payloads of therapeutics and deliver therapeutic levels of these drugs. Fourthly, the immobilization of the BDNF in the pores of the SPs serves to protect the protein from denaturing *in vivo* [53], and thus maintains its bioactivity [54] whereas protein instability over time is a problem when stored in mini-pumps [68]. This is important as neurotrophins have a relatively rapid half-life *in vivo* [68–70]. The results showing significant SGN survival support the conclusion that the released BDNF was biologically effective. Fifthly, the SP technology enables the use of multiple drugs, each loaded in separate SPs that can potentially have release characteristics tailored for best effect for each drug type. Finally, the SP system offers the clinician the option of implanting the SPs into the cochlea, for instance along with a cochlear implant, or implanting them onto to round window membrane (external to the cochlea). The round window membrane is permeable to small molecules and drugs [71, 72]. A current clinical approach to deliver therapeutic compounds to the cochlea to treat hearing loss is to inject drugs into the middle ear cavity and rely on passive diffusion of the drug across the round window membrane. However, with this technique much of the drug is quickly lost, thus limiting the efficacy of the technique for delivering drugs to the cochlea. Implantation of drug-loaded SPs onto the round window membrane would be expected to improve drug entry into the cochlea [49].

### Clinical Relevance for Cochlear Implants

Due to the progressive nature of many types of SNHL it is highly likely that delivery techniques providing localised drug delivery over extended durations will be most successful. Because the cochlea becomes accessible during implantation surgery, it provides the opportunity of implanting a therapeutic delivery device inside the cochlea, along with the electrode array. The features of the SP delivery system (as discussed above) make it a suitable drug delivery strategy for combination with cochlear implantation in order to improve nerve survival and/or to protect residual hearing.

There is a strong correlation in SGN survival and cochlear implant performance with greater numbers of surviving SGNs resulting in improved cochlear implant performance [16]. The development of clinically translatable neurotrophin delivery strategies that increase SGN survival and the survival of their peripheral fibres, and reduce the electrical thresholds [30, 41, 73] would therefore be likely lead to improvements in cochlear implant function in contemporary devices. Furthermore, ongoing SGN degeneration and cell loss is a significant impediment to the clinical implementation of current focussing strategies that aim to provide more precise neural activation within the cochlea [56, 74, 75]. Technology that prevents SGN and nerve fibre loss, and promotes SGN fibre regrowth, may improve the clinical outcomes of future stimulation strategies designed to improve the precision of neural activation within the cochlea.

## Conclusion

This study has shown that SPs can provide a safe and effective strategy to delivery therapeutics to the inner ear for SGN survival, with survival effects observed over a wide extent of the cochlea. The SPs were well tolerated by the cochlea providing evidence that the technique is clinically relevant. Future studies to evaluate the supraparticle distribution and the pharmacokinetics of the released therapeutic would pave the way for clinical translation of the drug delivery strategy. The technology could potentially be combined with a cochlear implant to improve implant performance.
